# Enhancing yield and nutritional quality of sweet potato through genotype selection and selenium application

**DOI:** 10.3389/fpls.2025.1639024

**Published:** 2025-07-31

**Authors:** Qing Liao, Ying Xing, Li-Ping Pan, Jin-Ping Chen, Yong-Xian Liu, Dong-Liang Huang

**Affiliations:** Guangxi Key Laboratory of Arable Land Conservation, Guangxi Key Laboratory of Sugarcane Genetic Improvement, Key Laboratory of Sugarcane Biotechnology and Genetic Improvement (Guangxi), Guangxi Academy of Agricultural Sciences, Nanning, China

**Keywords:** sweet potato, yield, quality, starch, selenium

## Abstract

Selenium (Se) is an essential micronutrient for human health, but its widespread deficiency remains a major public health concern worldwide. Biofortification of staple crops, such as sweet potato (Ipomoea batatas), offers a sustainable strategy to improve dietary Se intake. This study systematically evaluated the capacity for natural Se accumulation in 12 major local sweet potato varieties in Guangxi, China. In addition, the effects of different Se application methods and dosages, soil application (10 L/hm^2^ and 20 L/hm^2^), foliar spraying (1.5 L/hm^2^ and 3.0 L/hm^2^), and combined soil and foliar application, were investigated on yield and quality parameters in two representative varieties: Guiziweishu 1 (high Se accumulator) and Fushu 404 (low Se accumulator). Significant genotypic variation in Se accumulation was observed, with Guiziweishu 1 exhibiting the highest tuber Se content (0.0139 mg/kg), while Fushu 404 had the lowest (0.0030 mg/kg). However, none of the varieties met the local standard for Se-rich agricultural products (0.02–0.20 mg/kg), highlighting the need for exogenous Se supplementation. Field trials demonstrated that all Se application treatments significantly increased tuber Se content, with foliar and combined soil+foliar applications showing the greatest effectiveness. In Guiziweishu 1, all Se treatments except T1 achieved the Se-rich standard, whereas in Fushu 404, only T4 and T6 reached this threshold. Yield improvements were also observed, with the combined soil+foliar treatment (T6) resulting in the highest increases in both fresh yield (24.22% for Guiziweishu 1, 13.06% for Fushu 404) and dry tuber yield (36.52% and 25.77%, respectively), relative to the control group. Se application further enhanced starch and anthocyanin content in Guiziweishu 1, whereas the effects were less pronounced in Fushu 404. These findings underscore the importance of varietal selection and optimized agronomic practices for effective Se biofortification in sweet potato, providing a theoretical and practical basis for developing Se-riched sweet potato cultivation and contributing to improved crop quality, yield, and public health in Se-deficient regions.

## 1 Introduction

Selenium (Se) is a vital micronutrient for human health that plays a crucial role in numerous physiological processes, including antioxidant defense, thyroid hormone metabolism, and immune function (Rayman, 2012; Bai et al., 2024). As an essential trace element, Se is incorporated into selenoproteins, which are essential for mitigating oxidative stress and maintaining redox homeostasis at the cellular level (Hatfield et al., 2014; Elhodaky and Diamond, 2018). Despite its importance, Se cannot be synthesized by the human body and must be acquired through dietary intake, thereby rendering the Se content of food crops a critical determinant of human nutritional status (Combs, 2001; Toh et al., 2022).

Globally, Se deficiency is recognized as a significant public health concern, especially in regions characterized by low natural soils Se content, which, results in inadequate dietary intake (Haug et al., 2007). Chronic Se deficiency has been associated with numerous health disorders, including Keshan disease, Kashin-Beck disease, impaired immune response, and an increased risk of certain cancers (Tan et al., 2002; Vinceti et al., 2017; Shimada et al., 2021).

Se biofortification, the process of increasing the Se concentration in food crops, offers a sustainable solution to mitigate the risk of human Se deficiency (Schiavon et al., 2020). Agronomic Se biofortification has shown considerable promise in enhancing the Se content of staple foods, thereby improving human Se intake (Newman et al., 2019). However, the efficacy of Se biofortification depends on multiple factors, including soil Se availability, crop species, genotypic variation, and the method and timing of Se application (Hossain et al., 2021; Liao et al., 2024a, 2025).

Sweet potato (*Ipomoea batatas*) is a widely cultivated food crop, particularly in China, where it serves as a staple for millions and is valued for its adaptability, high yield, and its rich nutritional composition, including dietary fiber, carotenoids, vitamins, minerals, and linoleic acid (Liu et al., 2020; Escobar-Puentes et al., 2022; Cheng et al., 2025). Thus, the use of Se-rich sweet potato as a dietary source of Se is a promising strategy to mitigate the risk of Se deficiency. In recent years, sweet potato has gained increasing attention as a potential vehicle for Se biofortification, owing to its widespread cultivation and substantial dietary role (Mabeyo et al., 2015). However, the ability of sweet potato to accumulate Se varies considerably among cultivars, due to genetic and environmental influences (Hossain et al., 2021; Chen et al., 2022). Understanding the natural Se accumulation capacity of local sweet potato varieties is therefore essential for developing effective biofortification strategies. In addition, the development of variety-specific agronomic practice to enhance Se biofortification is both necessary and urgent.

Therefore, in soils with a total Se content of 0.42 mg/kg, which meets the Se-rich classification standards (DB 45/T 1442-2016, Classification Requirements for Total Selenium Content in Soil), this study first assessed the natural Se accumulation capacities of 12 major local sweet potato varieties in Guangxi, China. Two sweet potato varieties exhibiting significantly different Se accumulation characteristics were subsequently selected, and the effects of various Se application methods and dosages on sweet potato yield and quality were further investigated. This work provides a theoretical foundation for optimizing Se-riched sweet potato cultivation techniques, thereby improving sweet potato quality and yield, enhancing the competitiveness of agricultural products, and ultimately benefiting public health in Se-dificent regions.

## 2 Materials and methods

### 2.1 Materials

The experiment included twelve sweet potato varieties: Guifen 2, Guishu 6, Guijingshu 8, Guiziweishu 1, Sushu 16, Sushu 17, Fushu 404, Fushu 604, Guangshu 87, Shangshu 19, Ningzishu 1, and Longshu 31. The selenium (Se) fertilizer used was a self-formulated Se fertilizer in the form of amino acid-chelated solution, containing 0.88% selenium and 0.46% amino acids. The selenium source was analytical-grade sodium selenite (AR, 98% purity), purchased from Shandong Xiya Chemical Industry Co., Ltd. The amino acid raw material (45% concentration) was obtained from Shandong Jinshan Bioengineering Co., Ltd.

The field experiment was conducted at the Wuming Lijian Research Base of the Guangxi Academy of Agricultural Sciences, where the soil type was classified as red soil. The physicochemical properties of the test soil were determined as follows: pH, 5.98; organic matter, 9.45 g/kg; total nitrogen, 1.26 g/kg; total phosphorus, 0.45 g/kg; total potassium, 4.69 g/kg; alkali-hydrolyzable nitrogen, 47 mg/kg; available phosphorus, 9.7 mg/kg; available potassium, 122 mg/kg; and total Se, 0.42 mg/kg.

### 2.2 Evaluation of natural Se accumulation ability

The experiment was designed as a randomized block with three replicates. Each plot comprised four rows, each 4.5 m in length, with a ridge width of 1.0 m, covering a total area of 18 m². Plant spacing was maintained at 24–26 cm, resulting in a planting density of 40,000 plants per hectare. Sweet potatoes were cultivated following standard agronomic management practices. Harvesting was performed 4.5 months after planting.

Selenium content was determined using the hydride generation–atomic fluorescence spectrometry (HG-AFS) method (Liao et al., 2024b).The dried samples were ground into a fine powder, digested in HNO_3_-HClO_4_ (V/V=4:1), and reduced with 6 mol/L HCl. The Se content was qualified using hydride generation-atomic fluorescence spectroscopy (Jitian Instrument Co., Ltd, Beijing), in accordance with the “National Food Safety Standard-Determination of Selenium in Foods (GB 5009.93-2017)”.

### 2.3 Selenium application experiment

Two sweet potato varieties exhibiting significant differences in natural Se accumulation in their tubers were selected from the aforementioned set. The experiment included seven treatments, involving three Se application methods: soil application, foliar spraying, and combined soil + foliar spraying, each at two application levels, based on previous studies (Guo et al., 2016b; Hou et al., 2018; Pan et al., 2021). The control treatment (CK) received no Se fertilizer. Details of the seven treatments are presented in **Table 1**. A randomized block design with three replicates was employed. Each plot consisted of six rows, each 4.3 m in length, and 0.9 m in ridge width, covering a total area of 23.2 m². Plant spacing was maintained at 20 - 21 cm, resulting in a planting density of 45,000 plants per hectare. The Se solution was diluted with water and applied during the tuber formation stage of sweet potato growth. For soil application, the Se solution was evenly poured around the roots zone of the sweet potato plants. For foliar application, the Se solution was evenly sprayed onto the leaf surface of the sweet potato plants with a sprayer, ensuring that droplets formed and dripped off the leaves. In the treatments involving two foliar applications or a combination of soil and foliar application, the interval between applications was 10 days. All other cultivation practices followed standard agronomic procedures. Sweet potatoes were harvested 4.5 months after planting.

**Table 1:** Treatment of Se application.

Treatment	Application approach	Se solution dosage (L/hm^2^)	Amount of adding water (L/hm^2^)	Times of Se application (times)
T1	Soil application	10.0	4500	1
T2	Soil application	20.0	4500	1
T3	Spraying	1.5	900	2
T4	Spraying	3.0	900	2
T5	Soil application	10.0	4500	1
	Spraying	1.5	900	1
T6	Soil application	20.0	4500	1
	Spraying	1.5	900	1
CK	—	—	—	—

Se fertilizers were applied 2 times at 10 days’ interval on T3, T4, T5, T6 treatment.

### 2.4 Plant sampling and measurement

At harvest, all tubers from each plot were collected, and the fresh tuber yield was recorded and converted to yield per hectare. The tuber weight was measured using an electronic scale.

For dry weight determination, three tubers per plot were sampled, cut into 1 cm³ cubes, thoroughly mixed, and approximately 250 g of the mixture was dried at 60 °C until brittle, followed by drying at 100 °C until a constant weight was achieved. This procedure was repeated three times, and the average value was used for analysis. The dried tuber yield was calculated using the formula:

Dried tuber yield = fresh tuber yield × tuber dry matter content.

At harvest, samples of fresh tubers, stems, and leaves were also collected for quality analysis. Se content was measured using the hydride generation–atomic fluorescence spectrometry method as described in Section 2.2. Tuber dry matter content was determined following the method of Wang et al. (2012). Starch content was measured following the method of Wang et al. (1989), using the following formula:

Starch content (%) = dry matter content (%) × 0.86945 – 6.34587.

The anthocyanin content was determined using the pH 3.0 citrate–disodium hydrogen phosphate buffer method (Yang et al., 2013).

### 2.5 Statistical analysis

All statistical analyses were conducted using Microsoft Excel 2007 and SPSS version 19.0. One-way analysis of variance (ANOVA) was emplored to compare group mean, and Duncan’s multiple range test was subsequently applied to identify significant differences. Each experiment was conducted in triplicate, with data presented as means ± standard errors. Statistical significance was set at p < 0.05.

## 3 Results

### 3.1 Natural Se accumulation of different sweet potato varieties

Significant variation in Se content was observed among the 12 potato varieties. Guiziweishu 1 exhibited the highest tuber Se content (0.0139 mg/kg), while Fushu 404 had the lowest (0.0030 mg/kg), a 4.63-fold difference. In stems and leaves, Fushu 604 had the highest Se content (0.0275 mg/kg), whereas Shangshu 19 exhibited the lowest value (0.0069 mg/kg). Across all varieties, stems and leaves consistently showed higher Se accumulation than tubers at maturity. The considerable coefficient of variation observed in Se content among varieties suggests substantial genetic diversity in Se accumulation (**Table 2**).

**Table 2:** The Se accumulation ability of different sweet potato varieties.

Variety	Se content(mg/kg)	
	Root-tuber	Stem and leaf
Guifen 2	0.0133 ± 0.0008a	0.0171 ± 0.0008d
Guishu 6	0.0099 ± 0.0009b	0.0180 ± 0.0002c
Guijingshu 8	0.0095 ± 0.0008bc	0.0155 ± 0.0007e
Guiziweishu 1	0.0139 ± 0.0004a	0.0231 ± 0.0003b
Sushu16	0.0065 ± 0.0010d	0.0074 ± 0.0002f
Sushu 17	0.0036 ± 0.0001ef	0.0072 ± 0.0003fg
Fushu 404	0.0030 ± 0.0004f	0.0066 ± 0.0002g
Fushu 604	0.0069 ± 0.0003d	0.0275 ± 0.0004a
Guangshu 87	0.0042 ± 0.0004e	0.0076 ± 0.0010f
Shangshu 19	0.0034 ± 0.0003ef	0.0069 ± 0.0003fgf
Ningzishu 1	0.0085 ± 0.0006c	0.0161 ± 0.0005e
Longshu 31	0.0100 ± 0.0002b	0.0172 ± 0.0004d
Average	0.0077	0.0142
Variation coefficient	46.66	47.48

Different lowercase alphabets represented significant difference at 0.05 level.

The average tuber Se content at maturity was 0.0077 mg/kg. Notably, none of the varieties met the Guangxi local standard for Se-rich agricultural products (0.02–0.20 mg/kg), even Guiziweishu 1. This suggests that natural Se accumulation is inadequate to meet Se-enrichment standards, highlighting the need for exogenous Se application.

### 3.2 Effect of Se application on fresh tuber yield

Based on their natural Se accumulation capacity, Guiziweishu 1 and Fushu 404 were selected for Se-enrichment trials. All Se application treatments significantly increased fresh tuber yield in both varieties, with the T6 (combined soil+foliar application) treatment showing the greatest effect (**Table 3**).

**Table 3:** Effects of Se application on the yield of sweet potato.

Variety	Treatment	Fresh weight (t/hm^2^)	Dry matter rate(%)	Dry weight (t/hm^2^)	Compared to CK(%)	
					Fresh	Dry
Guiziweishu 1	T1	29.30 ± 1.34b	26.45 ± 0.12cd	7.75 ± 0.32cd	1.29	4.34
	T2	30.06 ± 1.40b	28.07 ± 0.20ab	8.44 ± 0.39bc	3.91	13.60
	T3	29.63 ± 3.40b	27.27 ± 0.93bc	8.06 ± 0.69bcd	2.41	8.51
	T4	29.58 ± 1.22b	25.77 ± 0.09d	7.62 ± 0.30cd	2.25	2.62
	T5	32.66 ± 2.77ab	26.94 ± 0.45c	8.79 ± 0.55b	12.89	18.30
	T6	35.94 ± 2.42a	28.21 ± 0.13a	10.14 ± 0.70a	24.22	36.52
	CK	28.93 ± 2.48b	25.66 ± 0.48d	7.43 ± 0.70d	—	—
Fushu 404	T1	28.34 ± 0.82b	21.32 ± 0.58abc	6.04 ± 0.31cd	0.27	6.05
	T2	28.92 ± 0.70ab	21.81 ± 1.62ab	6.31 ± 0.56bcd	2.33	10.77
	T3	31.36 ± 2.68ab	22.04 ± 0.42ab	6.91 ± 0.52ab	10.97	21.20
	T4	28.71 ± 1.40ab	20.70 ± 0.73bc	5.94 ± 0.35cd	1.56	4.26
	T5	30.32 ± 2.83ab	21.73 ± 0.91ab	6.57 ± 0.37abc	7.27	15.32
	T6	31.95 ± 0.67a	22.44 ± 0.90a	7.17 ± 0.19a	13.06	25.77
	CK	28.26 ± 1.934b	20.17 ± 0.33c	5.70 ± 0.34d	—	—

Different lowercase letters indicate significant differences (P<0.05).

**Figure 1:**
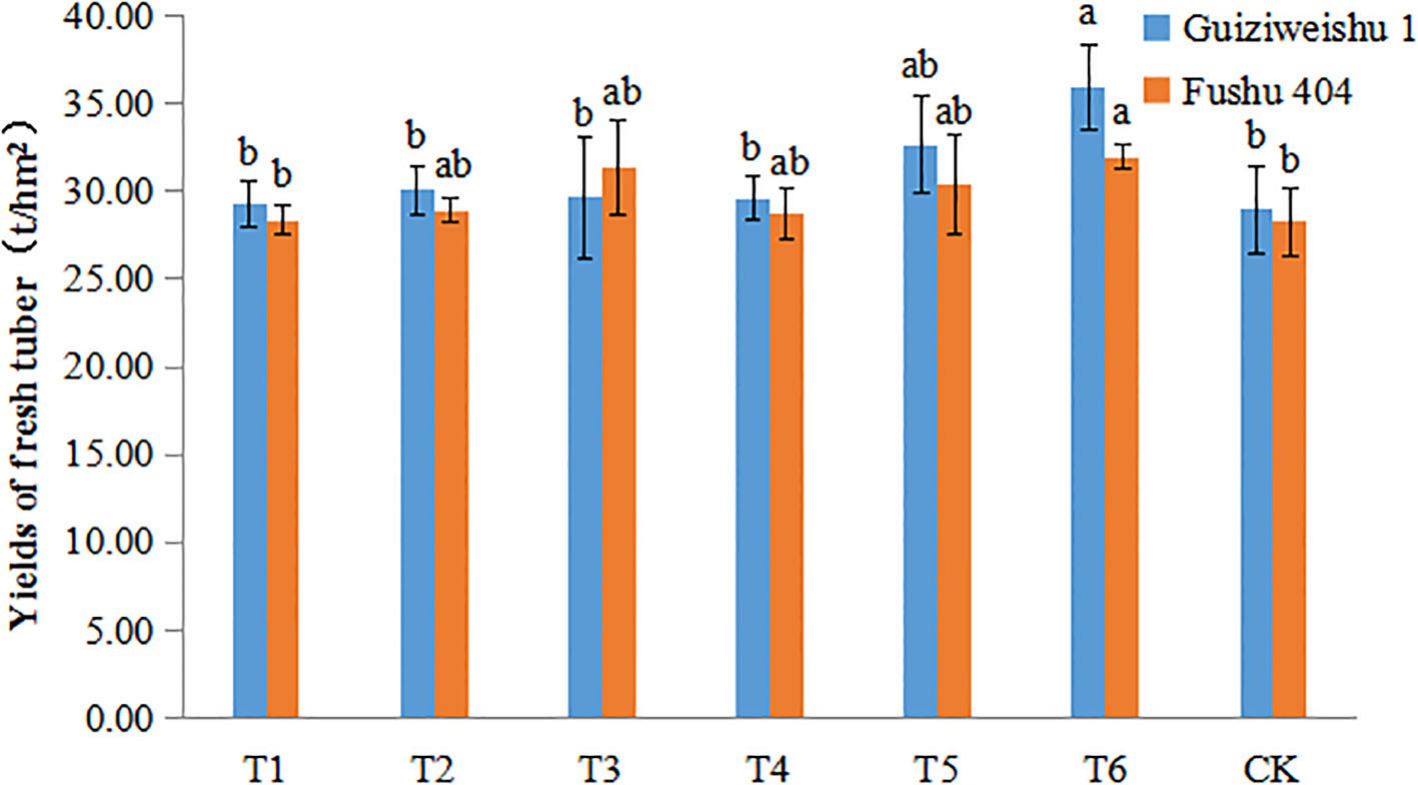
Fresh tuber yield of two sweet potato varieties under different Se application treatments. Different lowercase letters indicate significant differences (P<0.05).

For Guiziweishu 1, fresh tuber yield increased from 28.93 t/ha (CK) to 35.94 t/ha (T6), representing a significant increase of 24.22%. Other treatments resulted in yield increases of 1.29–12.89%, although these were not statistically significant (**Figure 1**; **Table 3**).

For Fushu 404, yield increased from 28.26 t/ha (CK) to 31.95 t/ha (T6), representing a significant increase of 13.06%. Other treatments produced yield increases of 0.27–10.97% that were statistically significant (**Figure 1**; **Table 3**).

### 3.3 Effects of Se application on dry tuber yield

Dry tuber yield was determined by adjusting the fresh tuber yield based on the dry matter content. Under various selenium (Se) application treatments, the dry tuber yield of both sweet potato varieties varied across treatments, with T6 producing the most pronounced improvement.

**Figure 2:**
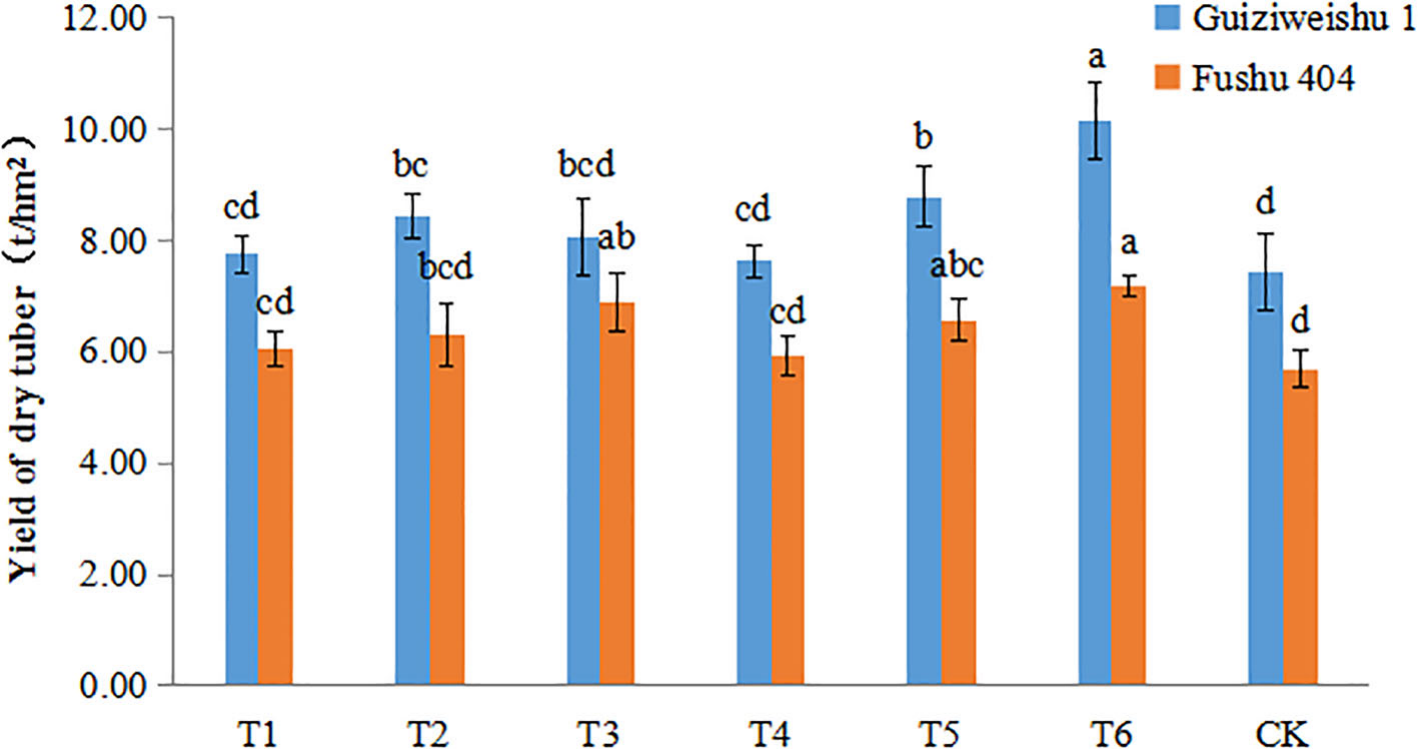
Dry tuber yield of two sweet potato varieties under different Se application treatments. Different lowercase letters indicate significant differences (P<0.05).

For Guiziweishu 1, the dry tuber yield under the CK treatment was 7.43 t/hm², whereas under T6, it increased to 10.14 t/hm², representing a statistically significant increase of 36.52%. The yields under T2 and T5 treatments also differed significantly from CK, while the other three Se treatments showed yield increases of 2.62% to 18.30%; however, these differences were not statistically significant compared to CK (**Figure 2**; **Table 3**).

For Fushu 404, the dry tuber yield under the CK treatment was 5.70 t/hm², and under T6 it reached 7.17 t/hm², a statistically significant increase of 25.77%. The T3 treatment yielded 6.91 t/hm², a significant increase of 21.20%. The T5 treatment resulted in a yield of 6.57 t/hm², a statistically significant increase of 15.32%. The remaining three treatments yielded increases ranging from 4.26% to 10.77%, but the differences were not statistically significant compared to CK (**Figure 2**; **Table 3**).

### 3.4 Effects of Se application on Se content in the tuber

**Figure 3:**
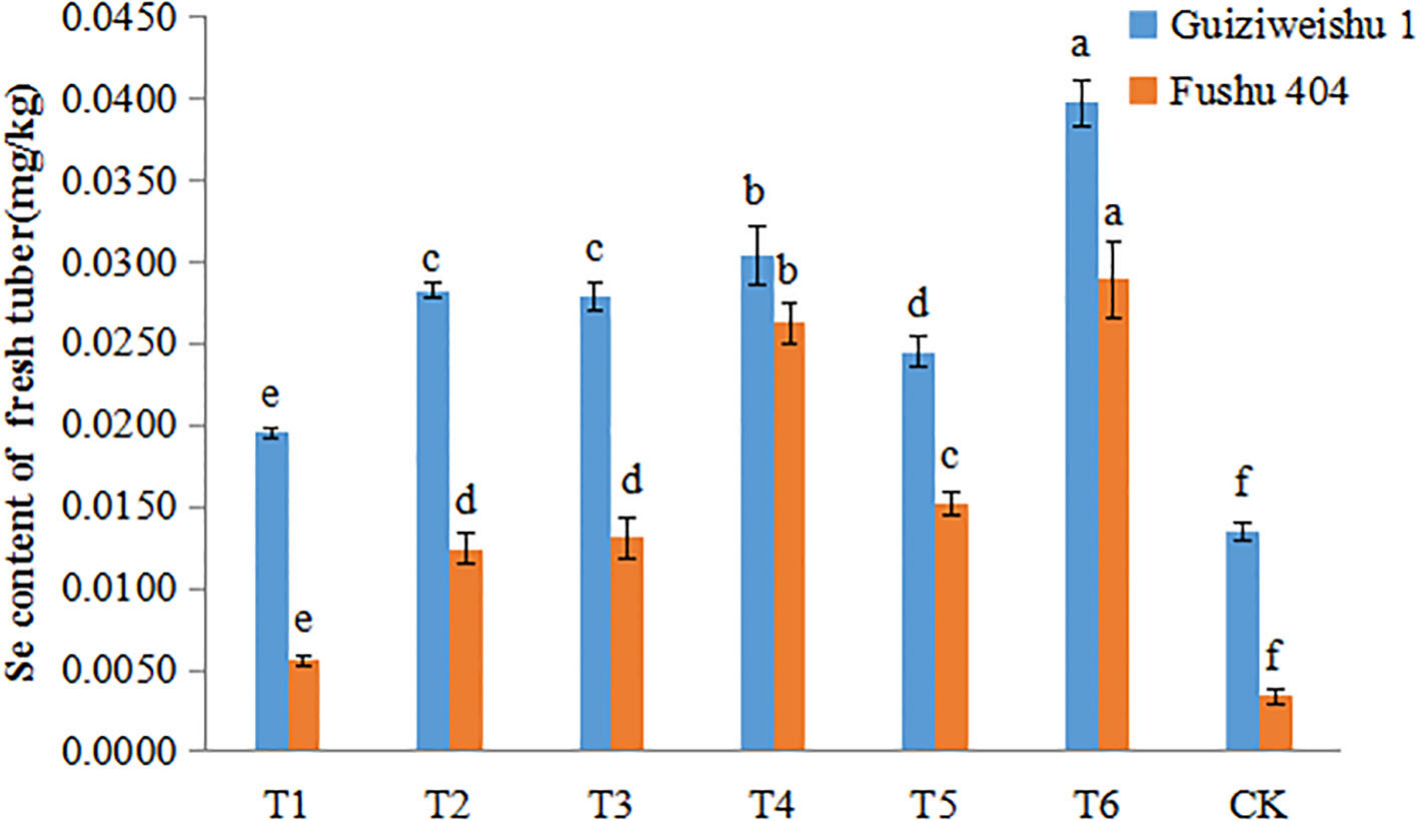
Selenium content of two sweet potato varieties under different Se application treatments. Different lowercase letters indicate significant differences (P<0.05).

In Guiziweishu 1, all Se treatments significantly increased the tuber Se content compared to the control (CK). In accordance with the local standard DB 45/T 1061-2014 for Se-riched agricultural products (sweet potatoes: 0.02–0.20 mg/kg), all Se treatments except T1 with a Se content of 0.0195 mg/kg, met the Se-rich threshold (**Figure 3**; **Table 4**).

**Table 4:** Effects of different Se application treatments on the quality of sweet potato tubers.

Variety	Treatment	Se-content (mg/kg)	Starch content (%)	Anthocyanin content (mg/100g FW)
**Guizhiweishu 1**	T1	0.0195 ± 0.0003e	16.65 ± 0.10cd	7.94 ± 0.46b
	T2	0.0282 ± 0.0005c	18.06 ± 0.17ab	9.50 ± 0.69a
	T3	0.0279 ± 0.0008c	17.36 ± 0.81bc	6.92 ± 0.23cd
	T4	0.0304 ± 0.0018b	16.06 ± 0.08d	9.27 ± 0.34a
	T5	0.0244 ± 0.0009d	17.08 ± 0.55c	7.61 ± 0.28bc
	T6	0.0397 ± 0.0015a	18.18 ± 0.11a	6.43 ± 0.30de
	CK	0.0134 ± 0.0006f	15.97 ± 0.42d	5.94 ± 0.09e
**Fushu 404**	T1	0.0056 ± 0.0003e	12.19 ± 0.51abc	16.92 ± 0.52bc
	T2	0.0124 ± 0.0009d	12.62 ± 1.41ab	17.82 ± 0.48ab
	T3	0.0131 ± 0.0012d	12.81 ± 0.36ab	16.63 ± 1.13c
	T4	0.0262 ± 0.0012b	11.65 ± 0.64bc	18.25 ± 0.51a
	T5	0.0152 ± 0.0008c	12.55 ± 0.79ab	16.88 ± 0.15bc
	T6	0.0289 ± 0.0023a	13.16 ± 0.78a	16.70 ± 0.70bc
	CK	0.0034 ± 0.0005f	11.19 ± 0.29c	17.75 ± 1.08abc

Different lowercase alphabets represented significant difference at 0.05 levels.

In Fushu 404, relative to the control (CK), all Se treatments also significantly elevated the tuber Se content. However, only T4 and T6 treatments met the Se-rich standard, with Se contents of 0.0262 mg/kg and 0.0289 mg/kg, respectively. The other four treatments failed to meet the required threshold (**Figure 3**; **Table 4**).

Compared to Fushu 404, Guiziweishu 1 demonstrated a greater capacity for Se accumulation, consistent with its greater inherent capacity for Se accumulation. Se application treatments significantly increased the Se content in sweet potato tubers, and the Se content exhibited a clear dose-response relationship with increased Se application rate. Notably, although the Se application rate in the foliar spray treatments was significantly lower than that in the soil application treatments, the spray treatments resulted in greater Se enrichment in the tubers. Furthermore, the Se content in tubers from the combined soil+foliar spray treatment was significantly higher than that from the soil-only treatment (**Figure 3**; **Table 4**).

### 3.5 Effects of Se application on starch content

**Figure 4:**
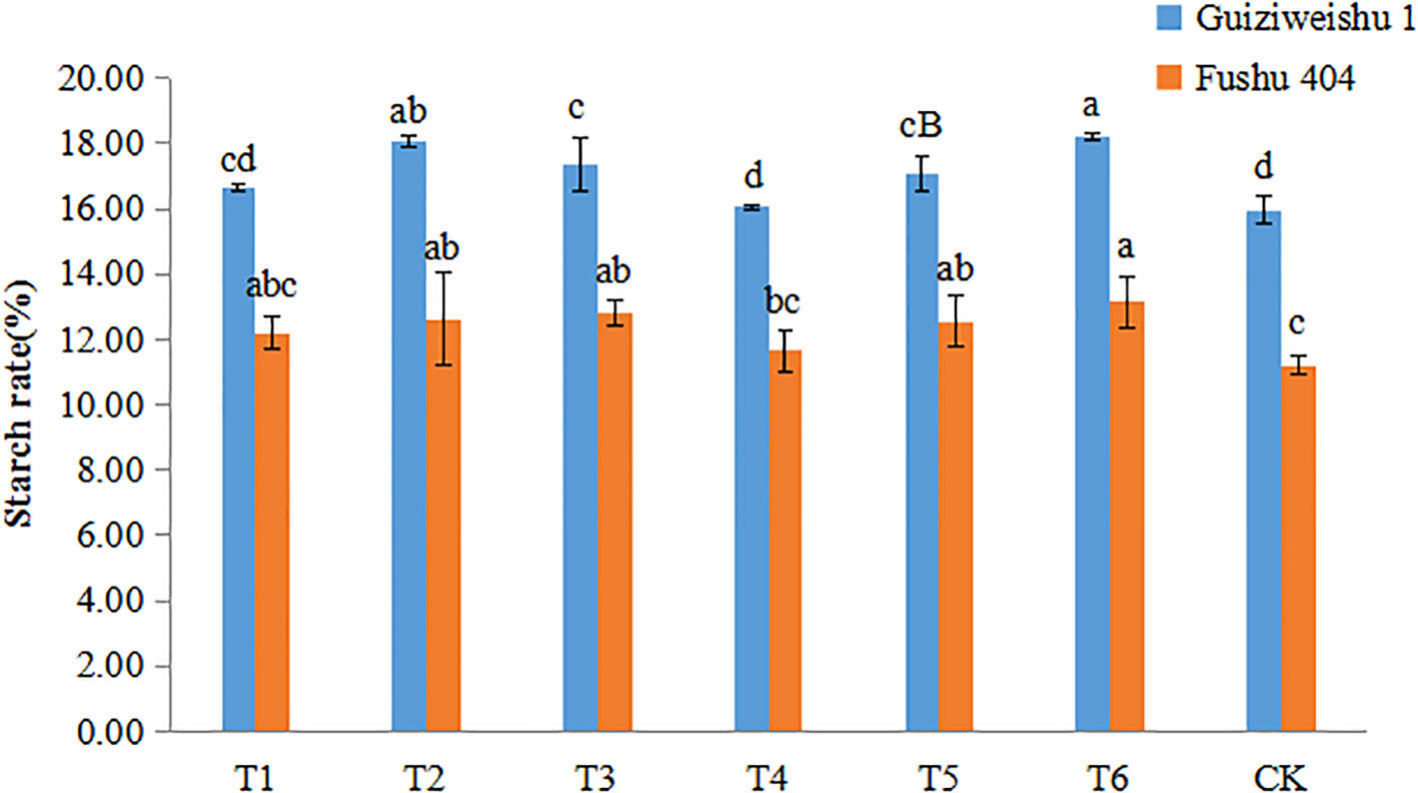
Starch content of two sweet potato varieties under different Se application treatments. Different lowercase letters indicate significant differences (P<0.05).

In Guiziweishu 1, compared to the control (CK), T2, T3, T5, and T6 significantly increased tuber starch content. T1 and T4 showed slight improvement, but the differences were not statistically significant. Among the soil Se application methods, T2 had a stronger effect on increasing starch content, exhibiting a significant difference compared to the other soil application treatment, T1. Within the foliar application treatments, T3 showed a greater increase in starch content than T4. A significant difference in starch content was also abserved between the two combined (soil + foliar) treatments, T5 and T6 (**Figure 4**; **Table 4**).

In Fushu 404, compared to the control (CK), all treatment significantly increased tuber starch content. However, no statistically significant differences were observed among the Se treatments except for T4. These results suggest that all Se application treatments significantly increase the starch content of Fushu 404 (**Figure 4**; **Table 4**).

### 3.6 Effects of Se application on anthocyanin content

**Figure 5:**
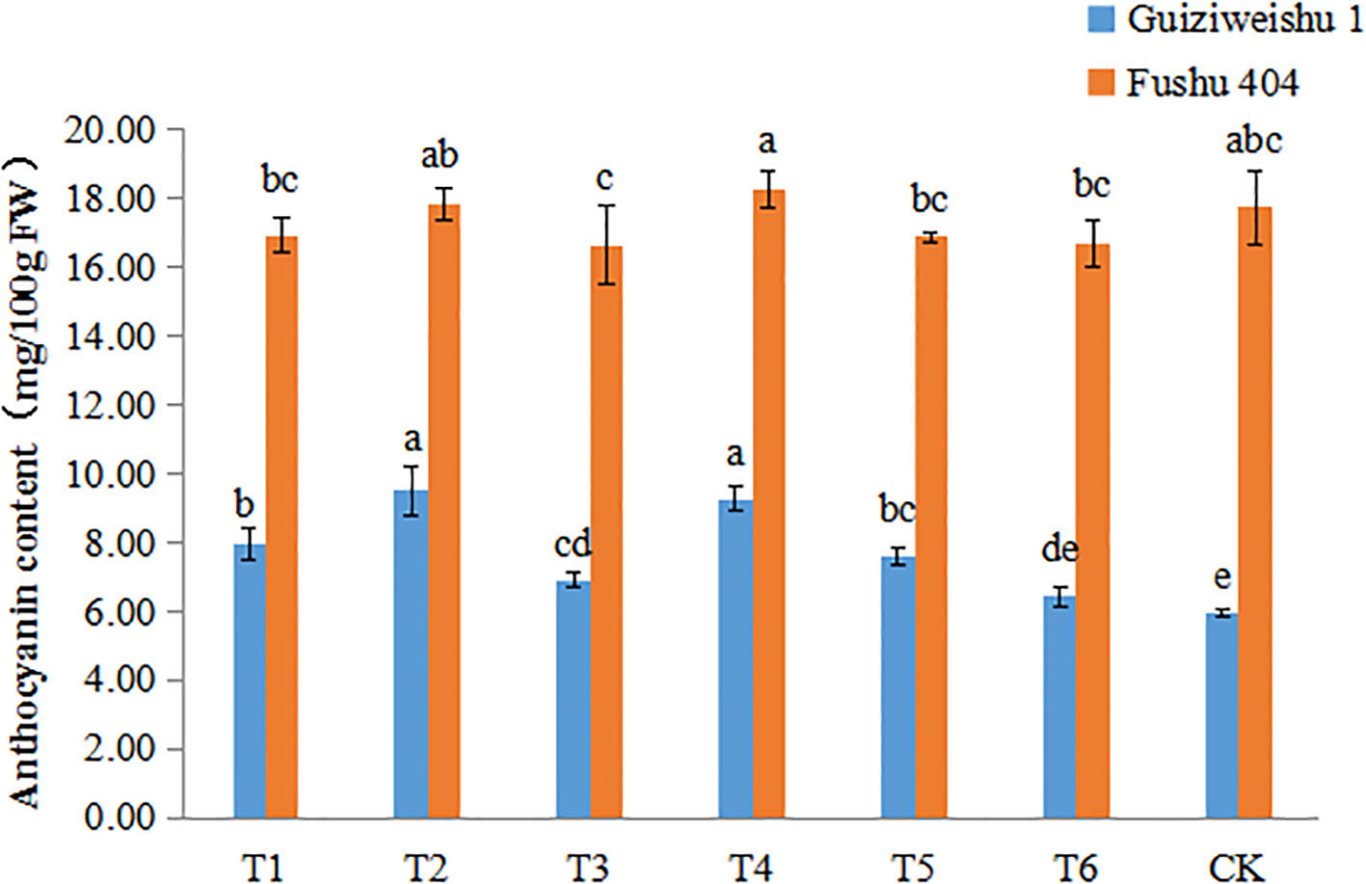
Anthocyanin content of two sweet potato varieties under different Se application treatments. Different lowercase letters indicate significant differences (P<0.05).

In Guiziweishu 1, all Se application treatments except for T6, significantly increased anthocyanin content compared to the control (CK). In contrast, in Fushu 404, no significant differences in anthocyanin content were observed among the Se treatments relative to the control (CK). These findings indicate that the impact of Se application on anthocyanin content was genotype-dependent (**Figure 5**; **Table 4**).

## 4 Discussion

Selenium (Se) is a vital micronutrient for human health that must be obtained through dietary intake. Sweet potato is a widely cultivated staple food crop. Supplying the human body with Se through Se-rich sweet potatoes is a feasible strategy to enhace Se intake. However, despite high soil Se content, Se concentration in crops exhibits significant variability, and often fail to meet established Se-rich standards (Liu et al., 2007; Wang et al., 2024). Genetic factors play a pivotal role in the uptake and accumulation of Se in crops (White, 2016; Dinh et al., 2018). Our study also revealed substantial genotypic variation in the natural Se accumulation abilities among 12 sweet potato varieties, with Guizhiweishu 1 exhibiting the highest Se content in tubers and Fushu 404 the lowest. The observed coefficient of variation in both tuber and stem/leaf Se concentrations underscores the diversity in Se uptake efficiency, which offers valuable potential for use in breeding programs targeting the development of biofortified sweet potato cultivars (Silva et al., 2022; Zhou et al., 2023).

However, despite this variation, even the highest naturally accumulated Se content (0.0139 mg/kg in Guizhiweishu 1 tubers) did not reach the Guangxi local standard for Se-rich agricultural products (0.02–0.20 mg/kg). The limited natural Se accumulation may be attributed to physiological barriers to Se uptake and translocation, as well as soil Se bioavailability (Hossain et al., 2021). These findings suggest that natural genetic variation alone is insufficient to meet Se-rich standards under typical field conditions, highlighting the need for exogenous Se fertilization.

Appropriate Se application has been shown to enhance crop growth, development, and yield (Chen et al., 2022; Luo et al., 2009; Wu et al., 2011; Zheng et al., 2013; Zhu et al., 2016). Our results demonstrated that exogenous Se application, particularly under the T6 treatment (soil + foliar application at high concentration), significantly increased both fresh and dry tuber yields in Guizhiweishu 1 and Fushu 404. The maximum yield increases were 24.22% and 13.06% in fresh tuber yield, and 36.52% and 25.77% in dry tuber yield for Guizhiweishu 1 and Fushu 404, respectively. The positive effect of Se on yield is multifaceted. At low to moderate concentrations, Se can mitigate oxidative stress, enhance photosynthetic efficiency, and mediate phytohormones in plants (Ikram et al., 2024). It may also stimulate the activity of antioxidant enzymes, thereby promoting plant growth and development (Lanz and Reis, 2021). However, the lack of significant yield increases under lower Se application rates (T1–T5) in some cases suggests a threshold effect, whereby only sufficient Se supplementation elicits pronounced growth responses. This is in line with the concept of Se as a “beneficial element” in higher plants, with a narrow margin between deficiency and toxicity (Gupta and Gupta, 2017).

The quality of Se-rich sweet potato is commonly assessed using indicators such as Se content, starch content, and anthocyanin content. Se application has proved to be an effective strategy for enhancing Se content in sweet potatoes (Li et al., 2008; Guo et al., 2016a; Li et al., 2021). This study demonstrated that Se fertilizers application significantly increased Se concentration in sweet potato tubers, exhibiting a clear positive correlation between the application rate and tuber Se concentration. Notably, foliar application, despite using lower Se dosages, was more effective than soil application, and the combined soil + foliar spray treatment (T6) resulted in the highest Se content. This finding aligns with previous reports indicating that foliar-applied Se is more efficiently absorbed and translocated to edible plant tissues than soil-applied Se, likely due to reduced soil Se fixation and enhanced direct foliar uptake (Wang et al., 2022; Hao et al., 2022). In Guizhiweishu 1, all treatments except T1 achieved Se concentrations that met the local standard for Se-rich products, whereas in Fushu 404, only T4 and T6 met the standard. This findings highlight the influence of genotype on Se uptake capacity, even under exogenous supplementation, consistant with observation in wheat (Boldrin et al., 2018; Ram et al., 2024). Moreover, the results suggest that, for varieties with inherently low Se accumulation capacity, higher application rates or more effective delivery methods (e.g., foliar spray) are necessary to achieve desired enrichment levels.

Se application also had significant effects on the nutritional quality of sweet potato tubers. In both varieties, all Se treatments led to a significant increase in starch content. This improvement may be attributed to Se-mediated enhancement of photosynthetic efficiency and the upregulation of key enzymes involved in starch biosynthesis (Malagoli et al., 2015; Ma, 2018). Notably starch content in Guiziweishu 1 was consistently higher than that in Fushu 404 across all treatments, suggesting that the impact of Se on nutritional quality is genotype-dependent (Pannico et al., 2019; Reis et al., 2020).

Anthocyanin content, another key nutritional attribute, was also influenced by Se application. In Guizhiweishu 1, all Se application treatments except T6, significantly increased anthocyanin content. The enhancement of anthocyanin synthesis by Se may be linked to its role in modulating antioxidant defense systems and the phenylpropanoid pathway (Reis et al., 2020; Ikram et al., 2024). In contrast, in Fushu 404, no significant differences in anthocyanin content were observed among the various Se treatments compared to the control. The differential responses between treatments and varieties suggest the involvement of complex regulatory mechanisms, potentially influenced by both environmental conditions and genetic background.

Our findings have several important implications for the development of Se biofortification strategies in sweet potato. First, although genetic variation in Se accumulation exists among varieties, natural Se levels in tubers remain insufficient to meet enrichment standards under typical field conditions, highlighting the necessity of exogenous Se supplementation. Second, both the method and rate of Se application are critical determinants of agronomic performance and nutritional quality. Foliar and combined soil + foliar applications are particularly effective in increasing tuber Se content and yield, as well as enhancing starch and anthocyanin levels. Thus, for varieties with inherently low Se accumulation capacity, higher Se application rates or more efficient delivery methods are required to achieve target enrichment levels. Conversely, varieties with higher Se accumulation capacity may achieve Se enrichment standards with lower Se inputs, thereby minimizing the risk of Se toxicity and reducing potential environmental impact.

While this study provides valuable insights into the effects of Se application on sweet potato yield and quality, several limitations should also be acknowledged. First, the study was conducted under specific environmental and soil conditions, which may influence Se bioavailability and plant response. Therefore, further research across diverse agroecological zones is necessary to validate the broad applicability of these findings. Second, although yield and key quality traits were assessed, the potential effects of Se application on other nutritional components, such as vitamins, minerals, sensory quality, and storage stability, remain unexplored and warrant further investigation. Finally, the long-term impacts of repeated Se application on soil health and environmental sustainability should be systematically evaluated to ensure safe and effective biofortification practices.

## 5 Conclusions

**Figure 6:**
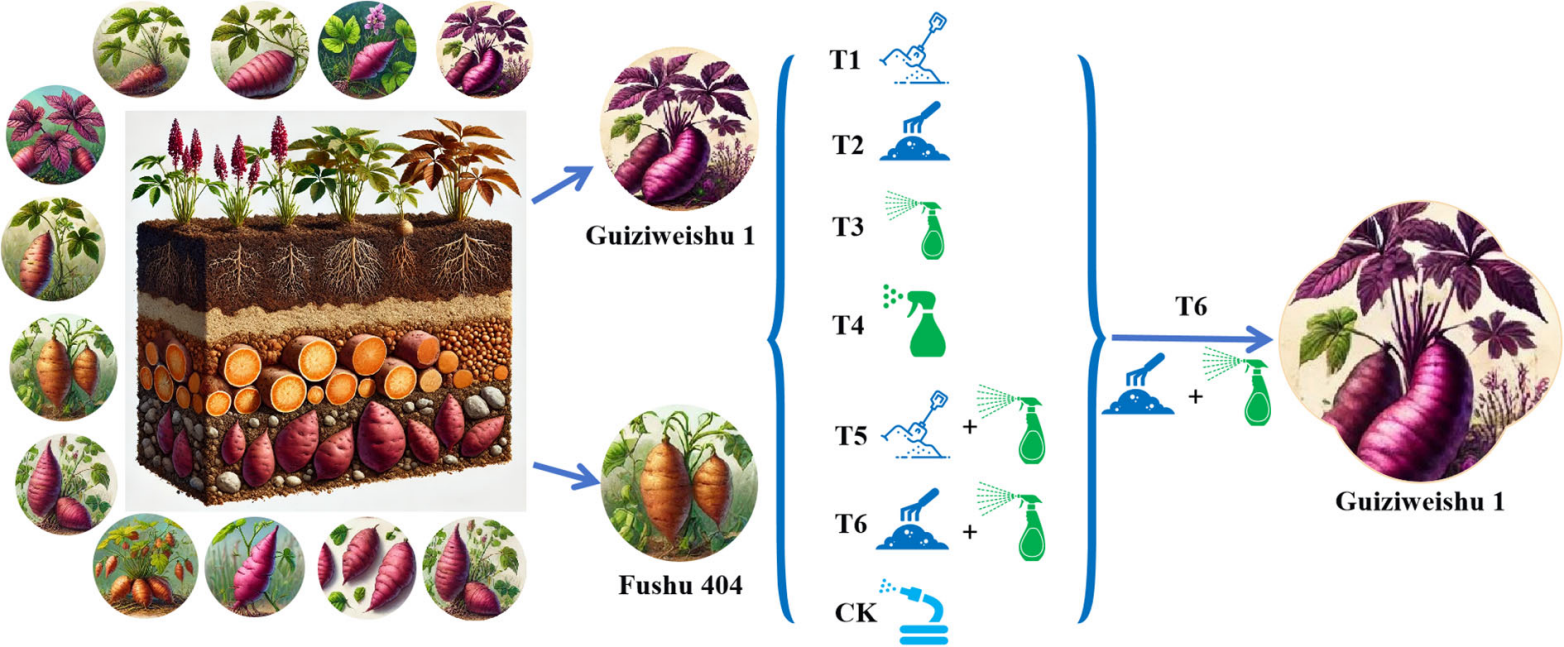
Strategy for enhancing Se accumulation of sweet potatoes.

Our study demonstrates that exogenous Se application, particularly through foliar or combined soil and foliar methods, significantly enhance Se content, yield, and nutritional quality in sweet potato tubers. The marked genotypic variation in Se accumulation capacity highlignts the importance of developing variety-specific biofortification strategies (**Figure 6**). These findings offer a scientific foundation for implementing practical Se biofortification programs in sweet potato cultivation, with potential benefits for improving human Se intake and promoting public health. Future research should focus on optimizing Se application protocols, elucidating the physiological and molecular mechanisms undelying Se uptake and metabolism, and evaluating the broader nutritional and environmental impacts of Se biofortification.

## Data Availability

The original contributions presented in the study are included in the article/supplementary material. Further inquiries can be directed to the corresponding authors.
